# Application of the pH-Responsive PCL/PEG-Nar Nanofiber Membrane in the Treatment of Osteoarthritis

**DOI:** 10.3389/fbioe.2022.859442

**Published:** 2022-04-27

**Authors:** Zetao Wang, Yanping Zhong, Si He, Ruiming Liang, Chuanan Liao, Li Zheng, Jinmin Zhao

**Affiliations:** ^1^ Guangxi Engineering Center in Biomedical Materials for Tissue and Organ Regeneration & Collaborative Innovation Center of Regenerative Medicine and Medical Biological Resources Development and Application, The First Affiliated Hospital of Guangxi Medical University, Nanning, China; ^2^ Department of Orthopaedics Trauma and Hand Surgery, Guangxi Key Laboratory of Regenerative Medicine, The First Affiliated Hospital of Guangxi Medical University, Guangxi Medical University, Nanning, China; ^3^ Life Sciences Institute, Guangxi Medical University, Nanning, China; ^4^ Postdoctoral Mobile Station of Clinical Medicine, Guangxi Medical University, Nanning, China

**Keywords:** osteoarthritis, naringenin, PCL, electrospinning, pH-responsive

## Abstract

Electrospinning technology is widely used in the field of drug delivery due to its advantages of convenience, high efficiency, and low cost. To investigate the therapeutic effect of naringenin (Nar) on osteoarthritis (OA), the pH-responsive system of the polycaprolactone/polyethylene glycol-naringenin (PCL/PEG-Nar) nanofiber membrane was designed and used as drug delivery systems (DDS) in the treatment of OA. The PEG-Nar conjugate was constructed via ester linkage between mPEG-COOH and the carboxyl group of naringenin, and the PCL/PEG-Nar nanofiber membrane was prepared by electrospinning technology. When placed in the weak acid OA microenvironment, the PCL/PEG-Nar nanofiber membrane can be cleverly “turned on” to continuously release Nar with anti-inflammatory effect to alleviate the severity of OA. In this study, the construction and the application of the pH-responsive PCL/PEG-Nar nanofiber membrane drug delivery platform would throw new light on OA treatment.

## Introduction

Osteoarthritis (OA) is a prevalent chronic degenerative disease of joint tissues and has been considered the most dominant reason for joint disability in elderly people (Hunter, 2011; Hunter et al., 2014; Katz et al., 2021). The typical symptoms of OA include pain, inflammation, cartilage loss, and decreased mobility, which seriously affect the patients’ quality of life. It is believed that intra-articular delivery of anti-inflammatory drugs is an effective method for OA treatment. However, currently available drugs for OA, such as nonsteroidal anti-inflammatory drugs (NSAIDs) and glucocorticoids, are considered to bring unwanted side effects to circulatory and digestive systems (Silverstein et al., 2000; Rodriguez-Merchan, 2018; Bannuru et al., 2019; Jones et al., 2019). Furthermore, the current therapeutic efficacy of the treatment of OA is not satisfactory, as many hydrophobic drugs have a short residence time in the body after injection into bone and joints, while repeated injections can cause additional damage to cartilage (Mcintyre et al., 2018; Nishiwaki et al., 2018; Harris et al., 2019; Lakin et al., 2019; Martinez-Orozco et al., 2019; Cibecchini et al., 2020; Matsuda et al., 2020). Therefore, exploiting substitutes with fewer side effects and exploiting intelligent drug delivery systems (DDS) to extend the residence time of the drug in the body would be important for OA therapy.

Naturally originated medicines are attracting more and more attention in recent years. As a naturally occurring flavonoid, naringenin (Nar) is known for its excellent anti-inflammatory, free radical scavenging, and antimicrobial effects. Recently, Nar has been found to show great potentials in the treatment of osteoarthritis (Zaragoza et al., 2020; Bailey et al., 2021; Berthelot et al., 2021; Glennie et al., 2021). Wang et al. (2017) reported that Nar can inhibit the NF-κB pathway and modulate matrix metalloproteinases, so it is thought to be a promising therapeutic option for alleviation of osteoarthritis. Manchope et al. (2018) evaluated the therapeutic effect of Nar on chronic arthritis induced by titanium dioxide in mice and found that it could effectively ameliorate OA by reducing oxidative stress and pro-inflammatory cytokines. Due to its hydrophobicity, Nar is administered orally in most cases. The low absorption by oral ingestion results in the poor bioavailability of Nar, which limits its application in the treatment of osteoarthritis. Developing new dosage forms for improving the solubility and bioavailability of Nar is highly necessary for the application of Nar in OA treatment.

Commonly employed traditional DDS mediums such as liposomes, micelles, nanoparticles, and hydrogels (Xie et al., 2017; Hou et al., 2018; Huang and Huang, 2018; Chen et al., 2020; Filipczak et al., 2020; Zhao et al., 2020; Zhu et al., 2021) can prolong the residence time of drugs but usually suffered from premature drug release. Recently, more and more researchers have begun to pay attention to stimuli-responsive DDS in recent years. The endogenous or exogenous stimuli include pH, redox conditions, temperature, and light (Maudens et al., 2018; Zerrillo et al., 2019; Arra et al., 2020; Liu et al., 2020; Wang et al., 2020; Zhao et al., 2021). As the occurrence of OA is accompanied by the change of the pH value of the inflammatory tissue microenvironment, pH-responsive DDS has been widely used in the treatment of OA (Xiao et al., 2010; Araujo et al., 2015). Jin et al. (2020) prepared a pH-responsive nanoparticle medicine with celastrol, hollow mesoporous silica nanoparticles (HMSNs), and chitosan for OA treatment. The intra-articular injectable system has greatly improved the solubility and the bioavailability of celastrol and effectively inhibited the articular surface erosion and joint effusion. Geng et al. (2021) fabricated a pH-responsive bisphosphonate-conjugated nano-apatite system and found that it can attenuate cartilage degeneration through suppressing the osteoclast activity and inhibiting subchondral bone abnormal remodeling. In our previous study, MMP-13/pH-responsive ferritin nanocages loaded with hydroxychloroquine, termed CMFn@HCQ, was designed for OA therapy (Chen et al., 2019). It was found that CMFn@HCQ nanocages could prolong the drug retention time to 14 days, thus alleviating osteoarthritis. It can be concluded that pH-responsive DDS would be an effective method for OA treatment.

**FIGURE 1:**
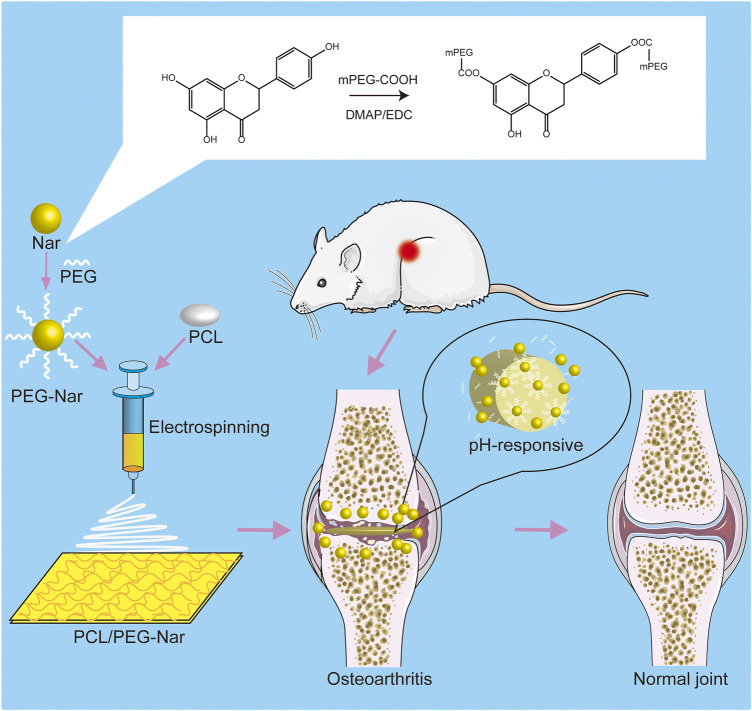
Schematic diagram of PCL/PEG-Nar nanofibrous membrane preparation and its treatment in OA.

In this study, a pH-responsive DDS made of a polycaprolactone/polyethylene glycol-naringenin (PCL/PEG-Nar) nanofiber membrane was prepared by electrospinning technology for OA treatment (Figure 1). Polyethylene glycol (PEG) with negligible toxicity and immunogenicity is approved by the FDA and has been widely applied in pharmaceutical preparation (Cao et al., 2021; Sreekanth et al., 2021), which promoted cell growth and improved drug solubility (Zhou et al., 2018). PCL is a biocompatible aliphatic poly-ester, which is commonly combined with drugs for electrospinning nanofibers (Chen et al., 2010; Li et al., 2020; Sencan et al., 2021). The PEG-Nar conjugate was constructed via ester linkage between mPEG-COOH and the carboxyl group of Nar. It is expected that Nar would be sustainably released through hydrolysis of the ester bond when the nanofiber membrane was placed in the weak acid inflammatory tissue microenvironment. The results based on ILβ-stimulated chondrocytes *in vitro* and an OA rat model *in vivo* were employed to verify the therapeutic effect of the pH-responsive system of the PCL/PEG-Nar nanofiber membrane on OA treatment. This study would offer a new strategy for increasing the solubility and bioavailability of natural product-derived drugs for OA treatment.

## Materials and Methods

### Synthesis and Characterization of Polyethylene Glycol-Naringenin

The synthesis of PEG-Nar was conducted based on a previous study (Eivazi et al., 2020; Xiong et al., 2021). Briefly, 1.0 g mPEG-COOH (Mw = 2000, Macklin, China), 0.5 g naringenin (Nar, J&K Scientific, China), 1.5 g 4-dimethylamino pyridine (DMAP, Sigma-Aldrich, United States), and 3.0 g 1-(3-dimethylaminopropyl)-3-ethyl carbodiimide hydrochloride (EDC, Sigma-Aldrich, United States) were dissolved in 1.0 ml dimethyl sulfoxide (DMSO, Macklin, China) under sonication for 5 min. All four solutions were mixed and stirred at 300 rpm (58°C, 8 h). Finally, the resulting solution was dialyzed in deionized water for 7 days, then lyophilized, and stored at 4°C.

The structure of PEG-Nar was characterized by ^1^H nuclear magnetic resonance spectroscopy (NMR), Fourier transform infrared spectroscopy (FTIR), and UV absorption spectrum. The ^1^H NMR spectrum of PEG-Nar was detected using a 400 MHz nuclear magnetic resonance spectrometer (AVANCE NEO 400) with DMSO*-d6* as a solvent. The FTIR was recorded using an FTIR spectrometer (PerkinElmer, Spectrum 100, United States) in the range of 400–4,000 cm^−1^. The PEG-Nar was investigated for UV absorption spectrum measurements. The UV absorption spectrum of the PEG-Nar was measured using a spectrophotometer (Lambda 650, China) within the range of 200–500 nm.

### Fabrication of pH-Responsive Electrospun Polycaprolactone/Polyethylene Glycol-Naringenin Nanofibrous Membranes

The pH-responsive electrospun PCL/PEG-Nar nanofibrous membranes were generated by the electrospinning method. In brief, 0.4 g polycaprolactone (PCL, Sigma-Aldrich, United States) was dissolved in 4.0 ml of hexafluoroisopropanol (HFIP, Macklin, China) and stirred at 300 rpm for 24 h. The PEG-Nar powder was added to 1.0 ml of HFIP and then stirred at 300 rpm for 6 h. The PEG-Nar and PCL solutions were mixed together with mass ratios of 1:99.5, 2:98, 4:97, 6:96, and 8:96, sonicated for 30 min and stirred for 24 h at 300 rpm. The concentration of PCL in HFIP was kept at 8 wt%. The mixed solution was prepared into nanofibrous membranes under 15.5 kV voltage, 0.8 ml/h dispensing rate, and 15 cm distance between the needle and the wheel. The electrostatic spinning process was performed at 25°C and 35–55% humidity. Finally, the PCL/PEG-Nar nanofibrous membranes were dried using a vacuum drying chamber (LGJ-10C, Foring Technology Development Co., Ltd., China). PCL/PEG-Nar with different contents of PEG-Nar (2, 4, 6, and 8%), PCL/PEG, and PCL/PEG/Nar nanofibrous membranes were obtained.

### Characterization of Electrospun Polycaprolactone/Polyethylene Glycol-Naringenin Nanofibrous Membranes

The morphology of PCL/PEG-Nar nanofibrous membranes was observed using a scanning electron microscope (SEM, Zeiss, SIGMA 500/VP, Germany). The nanofibrous membranes were treated with gold sputtering before observation. The average diameter of the fiber was tested based on the SEM images, and about 30 fiber diameters were analyzed with ImageJ software.

The mechanical capacity of the PCL/PEG-Nar nanofibrous membranes was measured using a universal testing machine (Instron 5,566, INSTRON, United States) at 6 mm/min crosshead speed. All nanofibrous membranes subjected to the mechanical test were cropped into a 30 mm × 10 mm rectangular shape.

The hydrophilicity of nanofibrous membranes was evaluated by the measurement of the static contact angles. The images were captured using a CCD camera (kgv-5000, Japan) and analyzed with the software program of the manufacturer.

To investigate the release of Nar from the nanofiber membrane, PCL/PEG-Nar and PCL/PEG/Nar nanofiber membrane were divided into four groups: 1) PCL/PEG/Nar nanofiber membrane, pH = 5.0 (inflammation environment); 2) PCL/PEG-Nar nanofiber membrane, pH = 5.0 (inflammation environment); 3) PCL/PEG/Nar nanofiber membrane, pH = 7.4 (normal environment); 4) PCL/PEG-Nar nanofiber membrane, pH = 7.4 (normal environment). A measure of 0.1 g nanofiber membranes was incubated in 20 ml phosphate-buffered saline (PBS) solution with different pH in an oscillator (37 °C). At each time point (0.5, 2, 4, 8, 16, 24, 48, 72, 96, 168, 336, 504, and 672 h), 2.0 ml release medium was collected, and 2.0 ml PBS solution was supplemented simultaneously. The Nar content was quantitatively detected with high-performance liquid chromatography (HPLC). The drug-releasing experiments were performed at least three times.

To evaluate the degradation, the nanofibrous membranes were incubated in PBS buffer under horizontal shaking (100 rpm, 37 °C) for 8 weeks, and the residuals (n = 3) were taken out, freeze-dried, and weighed in different periods of incubation.

### Cell Culture and Cell Seeding

Chondrocytes were extracted from the knee joints of 5-day-old Sprague–Dawley (SD) rats as in the previous study (Zerega et al., 2004; Deng et al., 2018). The performed experiment approach followed the standards of the Animal Ethics Committee of Guangxi Medical University. The isolated chondrocytes were cultured in a DMEM medium (Biosharp, China) with 10% fetal bovine serum (FBS, Tianhang, China) and 1% (v/v) streptomycin/penicillin (Biosharp, China). The chondrocytes were cultured in a cell incubator (Thermo Fisher Scientific, United States) at 5% CO_2_ and 37°C. The medium was exchanged every 3 days.

Before chondrocyte seeding, PCL/PEG-Nar nanofibrous membranes were cropped into 15-mm-diameter discs and then put into 24-well culture plates. The nanofibrous membranes were disinfected with 75% ethanol for 2 h and after that rinsed with PBS containing 1% streptomycin–penicillin. After drying, the specimens were disinfected under UV irradiation for 6 h. A total of 2×10^4^ chondrocytes were seeded in every 24-well culture plate. In total, five groups were divided: the chondrocytes were cultured on PCL/PEG, PCL/PEG/Nar, and PCL/PEG-Nar nanofiber membrane and then treated with 10 ng/ml IL-1β for 24 h, and the groups were designated as 1)normal group: chondrocytes without treatment, 2) OA group: chondrocytes induced by IL-1β, 3) PCL/PEG group: chondrocytes on the PCL/PEG nanofiber membrane induced by IL-1β, 4) PCL/PEG/Nar group: chondrocytes on the PCL/PEG/Nar nanofiber membrane induced by IL-1β, and 5) PCL/PEG-Nar group: chondrocytes on the PCL/PEG-Nar nanofiber membrane induced by IL-1β.

### Cell Proliferation and Viability Assay

The chondrocytes proliferation was evaluated by cell counting kit-8 (CCK-8, Biosharp, China) based on the product manual. The optical density was tested using a microplate reader (Thermo Fisher Scientific, United States) at 450 nm wavelength.

Cell viability was assessed by Calcein AM Cell Viability Assay Kit (Beyotime, China) according to the product manual. The chondrocytes cultured on nanofibrous membranes were incubated with a 1.0 μM PI and 1.0 μM calcein-AM for 5 min in the dark. The chondrocytes were observed after washing with PBS, and the images were captured using a laser scanning confocal microscope (Leica, TCS SP8). The chondrocyte morphology on the nanofibrous membranes was observed using SEM after 24-h culture.

### Quantitative Real-Time Polymerase Chain Reaction Assay

The gene expressions of matrix metalloprotein 3 (MMP3), matrix metalloprotein 13 (MMP13), interleukin-6 (IL-6), interleukin-1β (IL-1β), and collagen II (COL2a1) were conducted by quantitative real-time polymerase chain reaction (qRT-PCR) assay. The total RNA in chondrocytes was isolated using a HiPure Total RNA Mini kit (Magen, Guangzhou, China), and complementary DNA (cDNA) was synthesized with Prime Script™ RT kit (Takara, Japan). The qRT-PCR reaction was carried out using a real-time PCR system (LightCycler^®^480, Roche, Germany), and melting curve data were used to evaluate PCR specificity. The relative gene expression levels were analyzed by the 2^−ΔΔ CT^ method with glyceraldehyde-3-phosphate dehydrogenase (GAPDH) as a control. All specimens were repeated three times. The primers in the qRT-PCR reaction are shown in Table 1.

**TABLE 1:** Primers in qRT-PCR analysis.

Gene	Forward primer	Reverse primer
*GADPH*	CAC​GAC​ATA​CTC​AGC​ACC​AG	TCC​AGT​ATG​ACT​CTA​CCC​ACG
*MMP3*	GGC​TGT​GTG​CTC​ATC​CTA​CC	TGG​AAA​GGT​ACT​GAA​GCC​ACC
*MMP13*	GGA​CAA​AGA​CTA​TCC​CCG​CC	GGC​ATG​ACT​CTC​ACA​ATG​CG
*IL-1β*	GCA​CAG​TTC​CCC​AAC​TGG​TA	GCA​CAG​TTC​CCC​AAC​TGG​TA
*IL-6*	ACA​AGT​CCG​GAG​AGG​AGA​CT	ACA​GTG​CAT​CAT​CGC​TGT​TC
*COL2a1*	GTC​CTA​CAA​TGT​CAG​GGC​CA	ACC​CCT​CTC​TCC​CTT​GTC​AC

### Osteoarthritis Model and Treatment With the Polycaprolactone/Polyethylene Glycol-Naringenin Nanofiber Membrane

All animal procedures were performed based on the guidelines of the Animal Research Ethics Committee of Guangxi Medical University (Protocol Number: SCXK-Gui-2019–0011). A total of 30 SD rats (250 ± 30 g, aged 8 weeks, and 15 females and 15 males) were used in this study. The SD rats were fed in a controlled environment (25 ± 3°C, 40–60% relative humidity) and provided with food and water normally. The OA models were generated by anterior cruciate ligament transection (ACLT) as in previous studies (Setton et al., 1999; Stergiou et al., 2007). The nanofibrous membranes were cropped into 5 × 5 mm squares and disinfected with 75% ethanol for 2 h and after that rinsed with PBS containing 1% streptomycin–penicillin. After drying, the membranes were disinfected under UV irradiation for 6 h. The nanofibrous membranes were implanted and completely covered the cartilage surface of knee joints. Four weeks after surgery, the SD rats were anesthetized with sodium pentobarbital by intraperitoneal injection. A 1.5–2-cm sagittal incision was made at the joint to expose the joint cavity, and then the knee joints were covered with nanofiber membranes. In total, six groups were examined: 1) normal knees without any treatment, 2) normal knees treated with the PCL/PEG-Nar nanofiber membrane; 3) OA group with no treatment, 4) OA group treated with PCL/PEG nanofiber membrane, 5) OA group treated with the PCL/PEG/Nar nanofiber membrane; 6) OA group treated with the PCL/PEG-Nar nanofiber membrane. After 4-week treatment, the SD rats were euthanized, and the joints were collected for further examination.

### Macroscopic Observation and Histological Evaluation

Macroscopic observation of articular cartilage was performed by three independent observers, and the lesions of articular cartilage were graded based on the previous study (Strobel et al., 2003; Moskowitz, 2006), in which a higher score reveals more serious cartilage damage.

After macroscopic observation, the joints were fixed with 4% paraformaldehyde for 2 days and then decalcified. The joint tissues were embedded with paraffin and dissected into serial 3-μm sections. The histological examination was characterized by hematoxylin-eosin (H&E) staining and safranin O/fast green staining. The stained specimens were observed, and their images were captured using a microscope (BX53, Olympus, Japan). The Osteoarthritis Research Society International (OARSI) scoring system, an accepted standard for evaluating cartilage degeneration and damage (Mcalindon et al., 2014; Bannuru et al., 2019), was used to assess and grade the severity of the OA lesions.

### Statistical Analysis

The experimental results were expressed as mean ± standard deviation. All data were evaluated by one-way ANOVA and Tukey’s *t*-test with SPSS 21.0 software (IBM, United States). *p* < 0.05 indicated a significance level.

## Results

### Synthesis and Characterization of Polyethylene Glycol-Naringenin

**FIGURE 2:**
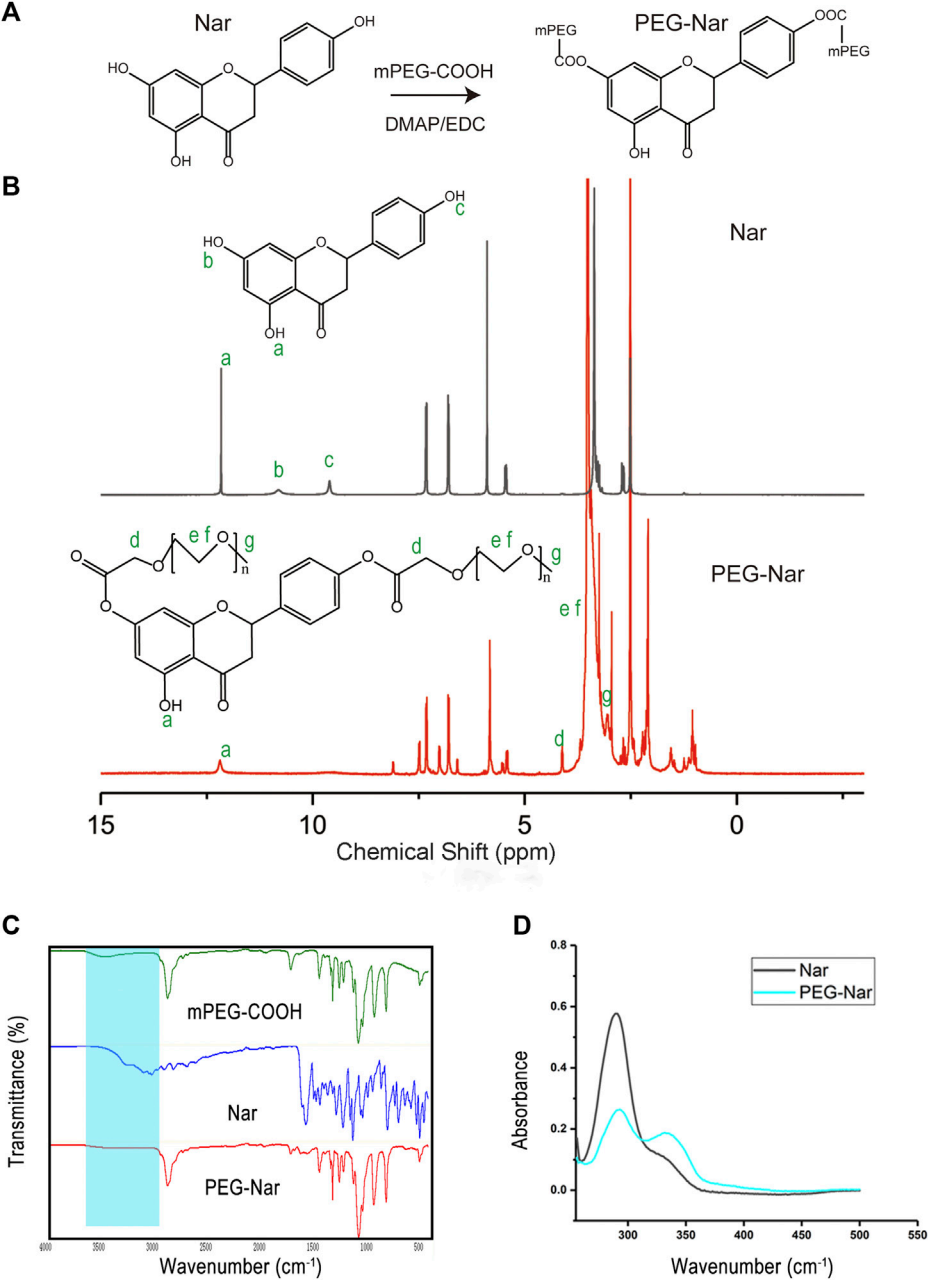
Synthesis and characterization of PEG-Nar. **(A)** Synthetic route of PEG-Nar. **(B)** 1H nuclear magnetic resonance spectroscopy (NMR) of PEG -Nar. **(C)** FTIR spectra of PEG-Nar and raw materials. **(D)** UV spectra of PEG-Nar and Nar.

As shown in Figure 2A, the PEG-Nar conjugate was synthesized by esterification of the carboxyl group of mPEG-COOH with the hydroxyl group of Nar under mild conditions. The ^1^H NMR results are shown in Figure 2B. The chemical shifts of Nar’s three Ar-OH and the corresponding proton numbers are *a* (12.16 (1H, s, Ar-OH), *b* (10.84 (1H, s, Ar-OH)), and *c* (9.60 (1H, s, Ar-OH)). In the NMR spectrum of PEG-Nar, the signals of *b* and *c* disappeared while that of *a* remained unchanged. In addition, PEG-Nar contains characteristic peaks of PEG (*d*, *e*, *f*, and *g*). According to the NMR results, it can be concluded that PEG-Nar was successfully synthesized, and PEG was grafted onto the *b* and *c* hydroxyl groups of Nar. FTIR and UV absorption spectrum were further employed to confirm the production of PEG-Nar. As shown in FTIR spectra (Figure 2C), the characteristic absorption peak of mPEG-COOH appeared at 1735 cm^−1^ (s; ν_as_(C=O)) and 3,300–2,500 cm^−1^(m; ν_as_(O-H)). The disappearance of the absorption peak of the hydroxyl group and the variation of the absorption peak of the carbonyl group indicate the successful synthesis of the PEG-Nar. From the UV absorption spectra of Nar (Figure 2D), the strong peak at 292 nm was ascribed to the characteristic band II of dihydroflavone compound, and the weak shoulder peak at 330 nm was ascribed to band I. The band I peak was greatly enhanced in the UV absorption spectra of PEG-Nar, indicating that mPEG-COOH is linked to Nar through the ester bond. The ^1^H NMR, FTIR, and UV results verified that PEG-Nar has been successfully synthesized.

### Characterization of the Nanofiber Membrane

**FIGURE 3:**
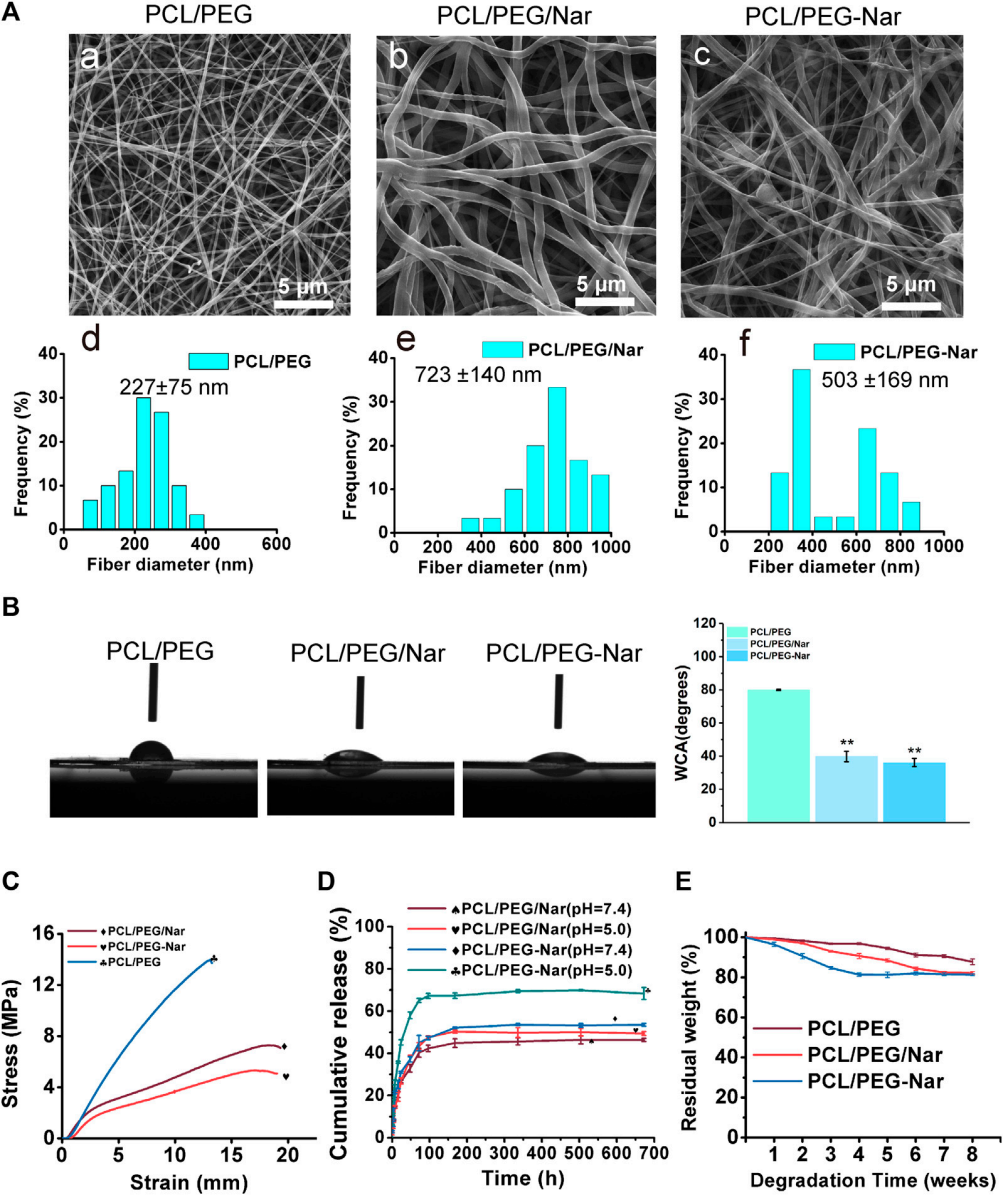
Characterizations of PCL/PEG, PCL/PEG/Nar, and PCL/PEG-Nar. **(A)** Scanning electron micrographs and diameter distribution of PCL/PEG **(a,d)**, PCL/PEG/Nar **(b,e)**, and PCL/PEG-Nar **(c,f)**. **(B)** Water contact angle of PCL/PEG, PCL/PEG/Nar, and PCL/PEG-Nar. ^*^ indicates *p* < 0.05, and ^**^ indicates *p* < 0.01. **(C)** Mechanical properties of PCL/PEG, PCL/PEG/Nar, and PCL/PEG-Nar. **(D)** Drug-releasing behavior of PCL/PEG/Nar and PCL/PEG-Nar. **(E)** Degradation rates of PCL/PEG, PCL/PEG/Nar, and PCL/PEG-Nar in PBS (pH 7.4) at 37°C.

The microstructure of the electrospinning nanofiber membrane was observed using SEM. The SEM images show that all nanofibers possessed a typical fiber scaffold with uniform structures and smooth surfaces, indicating that components of nanofiber were mixed evenly (Figure 3A). The average diameter of PCL/PEG, PCL/PEG/Nar, and PCL/PEG-Nar nanofibers are 227 ± 75 nm, 723 ± 140 nm, and 503 ± 169 nm, respectively. The average diameter of PCL/PEG/Nar and PCL/PEG-Nar nanofiber membranes was larger than that of the PCL/PEG nanofiber membrane, probably due to the addition of Nar decreasing the homogeneity of the electrostatic spinning solution. The average diameter of the PCL/PEG-Nar nanofiber membrane was smaller than that of the PCL/PEG/Nar nanofiber membrane, indicating that the homogeneity of the electrospinning solution was evidently improved when Nar bonding occurred with PEG.

The surface hydrophilicity of the nanofiber membrane was determined using an optical contact angle measurement system. As shown in Figure 3B, the contact angles of PCL/PEG, PCL/PEG/Nar, and PCL/PEG-Nar nanofiber membranes were 80.0 ± 0.34°, 39.8 ± 3.11°, and 36.1 ± 2.51°, respectively. The results showed that the contact angle decreased when Nar is added (*p* < 0.05). The small contact angle indicates that the hydrophilicity of the PCL/PEG-Nar nanofiber membrane was significantly improved and favorable for cell adhesion and growth (Martins et al., 2018; Orkwis et al., 2020).

The mechanical properties of PCL/PEG, PCL/PEG/Nar, and PCL/PEG-Nar nanofiber membranes were assessed by tensile testing, and the results are shown in Figure 3C and Table 2. Compared to those of the PCL/PEG nanofiber membrane, the tensile stress of the PCL/PEG/Nar nanofiber membrane decreases by 40.49%, the fracture strain increases by 47.72%, and the Young’s modulus decreases by 8.01%. In the case of PCL/PEG-Nar, the tensile stress decreases by 56.13%, the fracture strain increases by 48.38%, and the Young’s modulus decreases by 18.17%. The results indicate that the incorporation of PEG-Nar into PCL nanofiber can significantly reduce the stiffness but elevate the softness of the electrospinning membrane. The mechanical properties of the nanofiber membrane would be affected by the diameter of the nanofiber membrane (Heydari et al., 2017; Morel et al., 2018; Beckett et al., 2020); the addition of PEG-Nar increases the size of nanofibers and reduces the mechanical tensile stress and Young’s modulus of nanofibers.

**TABLE 2:** Mechanical properties of PCL/PEG, PCL/PEG/Nar, and PCL/PEG-Nar nanofiber membranes.

	Tensile stress (MPa)	Ultimate strain (%)	Young’s modulus (MPa)
PCL/PEG	12.47 ± 1.29	44.01 ± 0.57	42.96 ± 6.32
PCL/PEG/Nar	7.44 ± 0.31***	65.04 ± 0.74***	39.52 ± 2.34***
PCL/PEG-Nar	5.74 ± 0.48***	65.29 ± 4.31***	35.15 ± 6.48***

The drug-releasing behaviors of the PCL/PEG-Nar nanofiber membrane were investigated by HPLC. As shown in Figure 3D, the PCL/PEG/Nar nanofiber membrane reached the maximum in 168 h under the condition of pH = 7.4, and the cumulative release of Nar was 44.85% while that in the PCL/PEG-Nar nanofiber membrane was 50.28%. There was no significant difference between the two nanofiber membranes. On the contrary, the PCL/PEG-Nar nanofiber membrane reached the maximum release in 96 h under the condition of pH = 5.0, and the cumulative release of Nar was 69.43% while that was 52.10% in the PCL/PEG/Nar nanofiber membrane within 168 h. The results showed that the drug release rate of the PCL/PEG-Nar nanofiber membrane was significantly higher than that of the PCL/PEG/Nar nanofiber membrane (*p* < 0.05) under the acidic condition. Therefore, the pH responsiveness of PCL/PEG-Nar nanofiber membranes makes them suitable for controlled drug release.

The biodegradability of PCL/PEG, PCL/PEG/Nar, and PCL/PEG-Nar nanofiber membranes was investigated via weighing residues when incubating membranes in PBS buffer at 37 °C for 8 weeks. The results (Figure 3E) indicated that the PCL/PEG nanofiber membrane retained 87.76 ± 1.40% of the original weight after 8 weeks, and PCL/PEG/Nar and PCL/PEG-Nar nanofiber membranes retained 82.10 ± 0.72% and 81.32 ± 0.43%, respectively.

### Cell Proliferation

**FIGURE 4:**
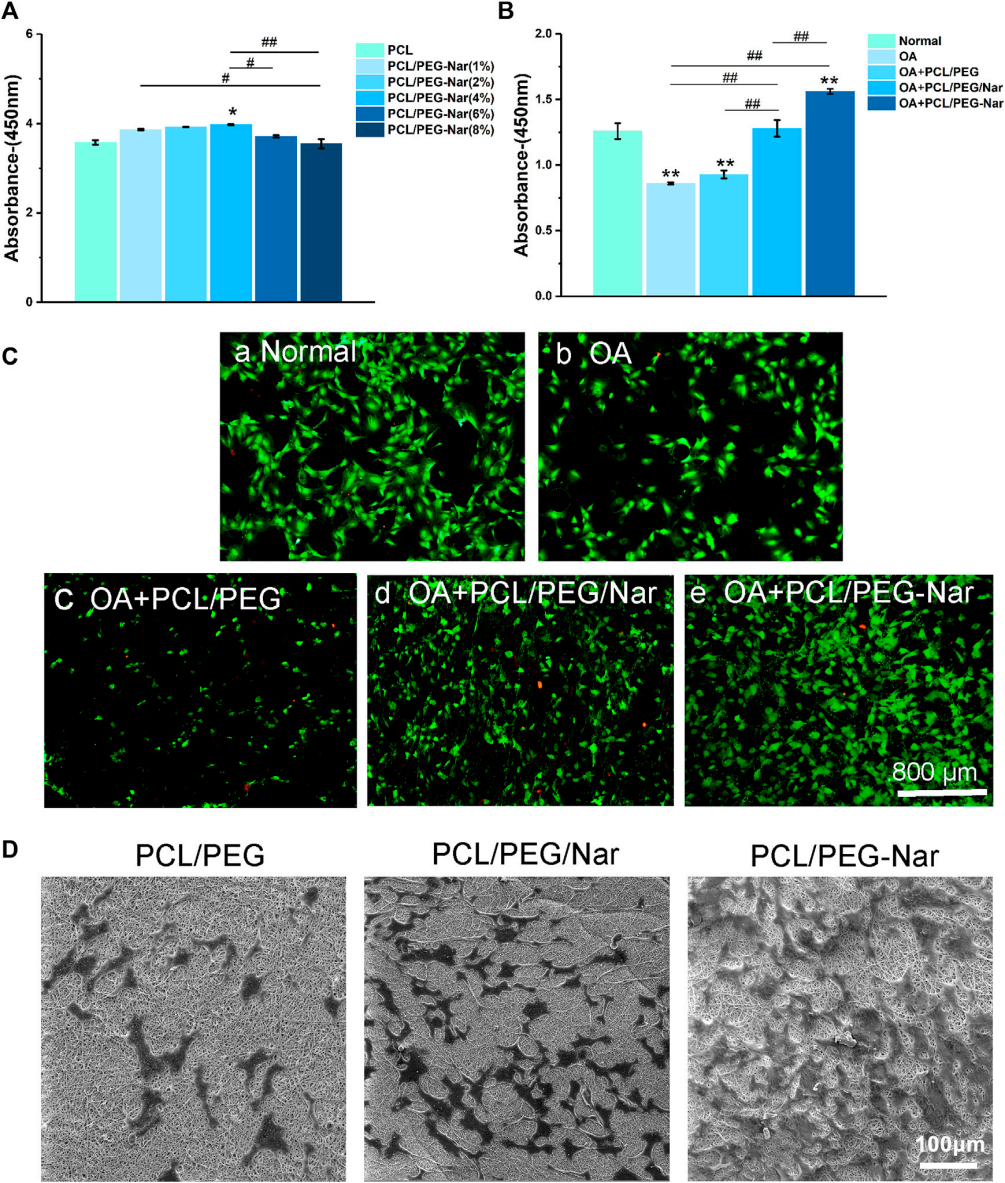
Cell proliferation and viability of IL-1β-stimulated chondrocytes on the nanofiber membrane. **(A)** Cytotoxicity of chondrocytes cultured on different concentrations of PCL/PEG-Nar. **(B)** Proliferation of chondrocytes on PCL/PEG, PCL/PEG/Nar, and PCL/PEG-Nar after treatment with IL-1β. **(C)** Cell viability of chondrocytes on PCL/PEG, PCL/PEG/Nar, and PCL/PEG-Nar after treatment with IL-1β. **(D)** Morphology of chondrocytes on PEG/Nar, PCL/PEG-Nar, and PCL/PEG membranes. ^*^ indicates *p* < 0.05, ^**^ indicates *p* < 0.01, and ^***^ indicates *p* < 0.001 compared with the normal group; ^#^ indicates *p* < 0.05, ^##^ indicates *p* < 0.01, and ^###^ indicates *p* < 0.001 with comparison between groups.

The influence of the nanofiber scaffold on cell proliferation was determined by CCK-8 assay, cell viability assay, and SEM observation. For the CCK-8 assay, the chondrocytes were cultured on nanofibrous membranes for 24 h. As shown in Figure 4A, cell proliferation is increased with the concentration of PEG-Nar when it is less than 4 wt%. However, cell proliferation will be decreased when the concentration of PEG-Nar was further increased to 6 wt% or higher. The results showed that PCL/PEG-Nar exhibited good biocompatibility, and the optimal concentration of PEG-Nar for the chondrocytes proliferation was 4 wt%.

The proliferation of chondrocytes on different nanofiber membranes after treatment with IL-1β was evaluated. It can be seen from Figure 4B that compared with that in the OA group, the proliferation of chondrocytes in the PCL/PEG/Nar nanofiber membrane group and PCL/PEG-Nar nanofiber membrane group was increased by about 49.4 and 83.5%, respectively. The results showed that the PCL/PEG-Nar nanofiber membrane showed the best biocompatibility among all these nanofiber membranes.

The viability of chondrocytes on the nanofiber membrane was further detected by calcein-AM/PI staining. As shown in Figure 4C, there are more living cells (green) and less death (red) in the PCL/PEG-Nar group than those in the other groups. The results confirmed that the PCL/PEG-Nar nanofiber membrane supports chondrocyte proliferation effectively.

Last, cell morphology on PCL/PEG, PCL/PEG/Nar, and PCL/PEG-Nar was observed using SEM after 24 h culture. As shown in Figure 4D, the number of cells on the PCL/PEG nanofiber membrane was lower than that on PCL/PEG/Nar and PCL/PEG-Nar nanofiber membranes, indicating that Nar-incorporated nanofiber membranes have excellent biocompatibility and proliferation effect on chondrocytes. In addition, chondrocytes spread out smoothly and almost fused on the PCL/PEG-Nar nanofiber membrane. The cell number of the PCL/PEG-Nar nanofiber membrane was significantly higher than that of the PCL/PEG/Nar nanofiber membrane. The results indicated that the PCL/PEG-Nar nanofiber membrane can increase cell adhesion and is the most suitable for chondrocyte proliferation.

### Quantitative Real-Time Polymerase Chain Reaction Analysis

**FIGURE 5:**
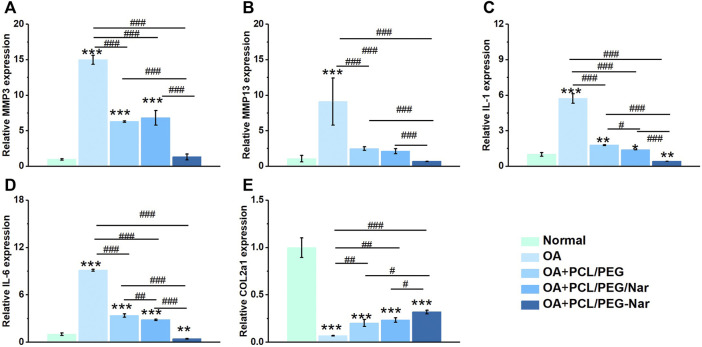
Gene expressions of **(A)** MMP3, **(B)** MMP13, **(C)** IL-1, **(D)** IL-6, and **(E)** COL2a1 of chondrocytes on PCL/PEG-Nar nanofibers after treatment with IL-1β. * indicates *p* < 0.05, ** indicates *p* < 0.01, and *** indicates *p* < 0.001 compared with the normal group; ^#^ indicates *p* < 0.05, ^##^ indicates *p* < 0.01, and ^###^ indicates *p* < 0.001 with comparison between groups.

To confirm the anti-inflammatory activity of nanofiber membranes on IL-1β-induced chondrocytes, qRT-PCR was conducted to detect the expression level of inflammatory genes and cartilage-related genes in chondrocytes, and the results are shown in Figure 5. The expression levels of inflammation-related genes were all downregulated and the cartilage-related genes were upregulated when chondrocytes were co-cultured with nanofiber membranes. Furthermore, when compared with the OA group, the PCL/PEG-Nar group downregulated MMP3 by 91.26%, MMP13 by 92.43%, IL-1 by 92.84%, IL-6 by 95.17%, and upregulated COL2a1 by 416.67%; the PCL/PEG/Nar group could downregulate MMP3 by 54.53%, MMP13 by 76.75%, IL-1 by 75.74%, IL-6 by 68.96%, and upregulated COL2a1 by 283.33%. The results showed that the PCL/PEG-Nar nanofiber membrane had a stronger anti-inflammatory ability than the PCL/PEG/Nar nanofiber membrane.

### Polycaprolactone/Polyethylene Glycol-Naringenin Nanofiber Membrane Attenuating Osteoarthritis *In Vivo*

**FIGURE 6:**
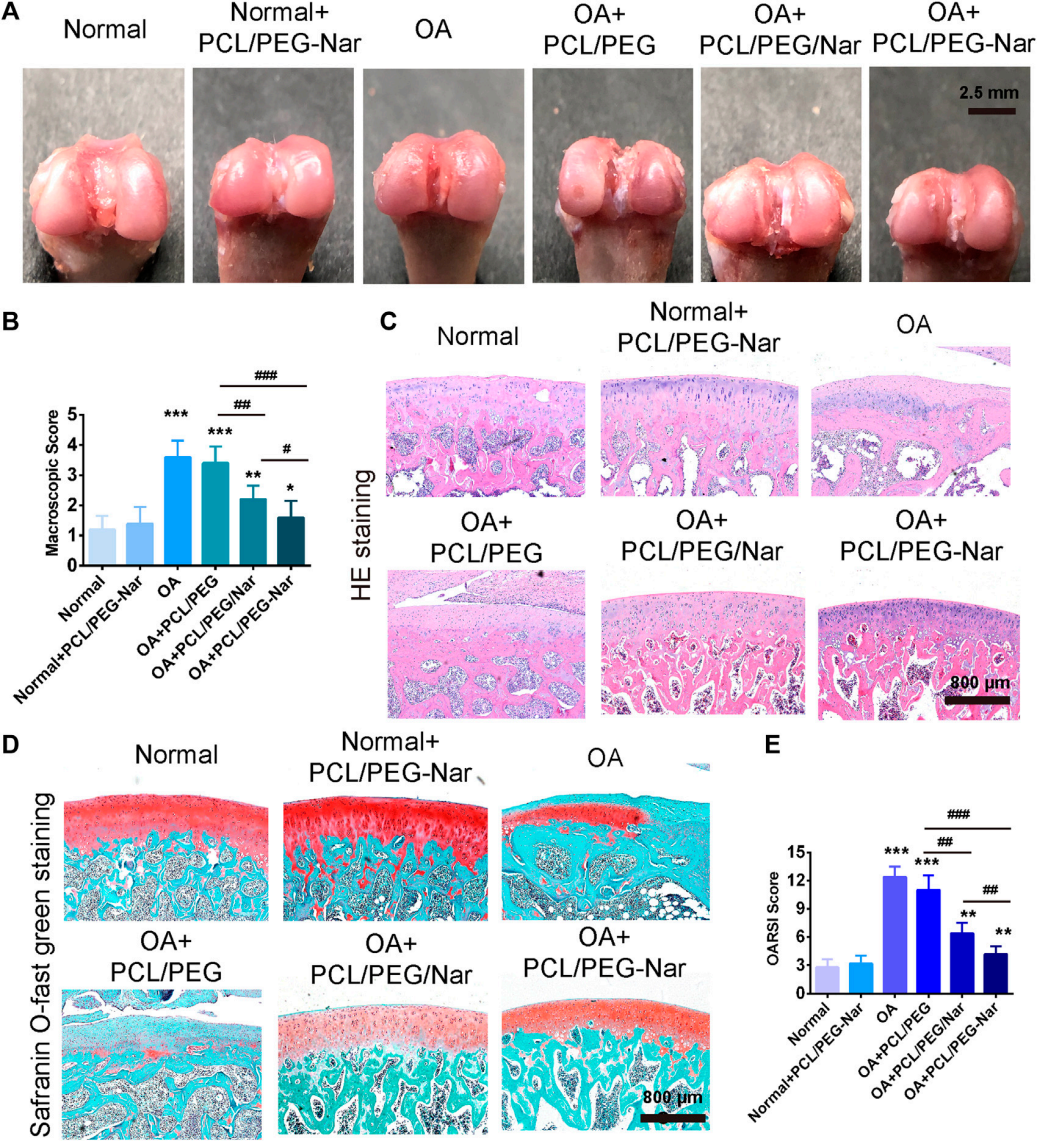
Effect of PCL/PEG-Nar on attenuating OA progression *in vivo*. The macroscopic observation **(A)** and macroscopic score **(B)** of cartilage after treatment with the PCL/PEG-Nar nanofibrousmembrane for 4 weeks. **(C,D)** H&E staining and safranin O/fast green staining. Scale bar: 800 μm. **(E)** OARSI score to evaluate cartilage degeneration and damage after PCL/PEG-Nar nanofibrous membrane therapy for 4 weeks. Mean ± SD; n = 3; ^*^ indicates *p* < 0.05, ^**^ indicates *p* < 0.01, and ^***^ indicates *p* < 0.001 compared with the normal group; # indicates *p* < 0.05, ^##^ indicates *p* < 0.01, and ^###^ indicates *p* < 0.001 with comparison between groups.

To investigate the therapeutical effect of PCL/PEG-Nar on attenuating OA progression *in vivo*, PCL/PEG, PCL/PEG/Nar, and PCL/PEG-Nar nanofibrous membranes were implanted to repair the injured cartilaginous tissue of OA SD rats in 4-week therapy. As shown in Figure 6A, macroscopic observation to assess degenerated cartilage indicated that there was no adverse effect on cartilage after PCL/PEG-Nar nanofibrous membrane treatment. The glistening and smooth surface cartilage in the joint was observed, which was similar to that in the normal group. Osteoarthritis pathological features, such as osteophyte formation, large lesions, and erosion, were observed in the femoral condyles in the OA group and the PCL/PEG treatment group. The cartilage defects in the PCL/PEG/Nar group were also obvious. After the treatment of the PCL/PEG-Nar nanofibrous membrane, cartilage damage was markedly reduced and recovered well with the glistening and smooth surfaces. A macroscopic score was also used to evaluate cartilage injury (Figure 6B). The OA joints treated with the PCL/PEG-Nar nanofibrous membrane had the lowest macroscopic score (1.60 ± 0.31) among all of the treatment groups, with a 55.56% decrease as compared with the OA group.

Histological analyses with HE and safranin O/fast green staining were further performed to determine cartilage degeneration. As shown in Figure 6C and Figure 6D, pronounced morphological and pathological features, such as fissures and fibrillation, osteophyte proliferation, and glycosaminoglycan (GAG) loss, were shown in OA and PCL/PEG groups. Morphological improvements, such as significant step-down cartilage degeneration, a remarkable reduction in the severity of histologic lesions, and proteoglycan retention, were demonstrated by the treatment of PCL/PEG/Nar and PCL/PEG-Nar after 4 weeks. In both groups, cartilage surface injury was most effectively recovered by PCL/PEG-Nar therapy, with the smoothest and integrated cartilaginous structure and increased GAG content. Furthermore, the OARSI score was also used to evaluate cartilage damage and degeneration (Figure 6E). Relative to the OA group, PCL/PEG/Nar and PCL/PEG-Nar treatment reduced the OARSI score by 48.38% and 77.42%, respectively, which is consistent with the macroscopic evaluations.

## Discussion

Due to the acidic microenvironment of the OA joint, the pH-responsive DDS is thought to be an effective method for OA treatment. For improving the solubility and bioavailability of the naturally occurring Nar with an anti-inflammatory effect, we constructed a pH-sensitive DDS made of a PCL/PEG-Nar nanofiber membrane by electrospinning technology and verified its therapeutic effect on the treatment of OA through *in vitro* and *in vivo* experiments.

Incorporation of the PEG-Nar conjugate into PCL nanofiber modulated the structures and physicochemical properties of the nanofiber membranes. The PEG-Nar conjugate was successfully synthesized by mPEG-COOH and Nar through the esterification reaction, as confirmed by ^1^H NMR, FTIR, and UV spectroscopy (Figures 2B–D). Through electrospinning, the PEG-Nar conjugate was incorporated into PCL, and the PCL/PEG-Nar nanofiber membranes were prepared. The addition of Nar decreases the homogeneity of the electrospinning solution, but the situation was improved when the Nar bonding occurred with PEG instead of direct blending. As a result, the average diameter of the PCL/PEG-Nar nanofiber membrane was smaller and the specific surface area was higher than those of the PCL/PEG/Nar nanofiber membrane, making it conducive to drug release. The mechanical test revealed that the blending of PEG-Nar increased the softness of the electrospinning membrane. In addition, the incorporation of PEG-Nar into the PCL matrix obviously improved the hydrophilicity of nanofiber membranes. Furthermore, the drug release rate of the PCL/PEG-Nar nanofiber membrane was significantly higher than that of the PCL/PEG/Nar nanofiber membrane in a weak acid environment (Figure 3D), indicating that the ester bond of the PEG-Nar conjugate endowed PCL/PEG-Nar nanofiber membranes with the ability of pH-responsive drug delivery. It is reported that electrospinning nanofiber with high porosity and hydrophilicity, moderate stiffness, and the diameter was favorable for the growth of cartilage (Chakraborty et al., 2009; Cheng et al., 2018; Aidana et al., 2021). The PCL/PEG-Nar nanofiber membranes exhibited desirable physicochemical properties, making them suitable for cartilage tissue regeneration and nanosustained-release system.

The PCL/PEG-Nar nanofiber membrane showed good affinity and biocompatibility to chondrocytes, such as improving cell viability and low cytotoxicity (Figures 4A–C). Cells grew better on the PCL/PEG-Nar nanofiber membrane than on the PCL/PEG/Nar nanofiber membrane. SEM results showed that chondrocytes were spherical cartilage on the PCL/PEG-Nar nanofiber membrane without deformation (Figure 4D), which was a typical phenotype of chondrocytes. Previous studies have shown that Nar can stimulate biological function and inhibit acetylcholine esterase activity, oxidative stress, and proinflammatory cytokine release (Alam et al., 2014; Chtourou et al., 2016; Tvrda et al., 2020). In this study, the anti-inflammatory ability of the PCL/PEG-Nar nanofiber membrane was better than that of the PCL/PEG/Nar nanofiber membrane alone. The expression of inflammatory genes, including *MMP3, MMP13, IL-1, and IL-6*, was inhibited in the PCL/PEG-Nar group. In addition, the PCL/PEG-Nar nanofiber membrane also promoted the expression of COL2a1 (Figure 5E). The results of *in vivo* experiment further confirmed the curing effect of the PCL/PEG-Nar nanofiber membrane. From macroscopic observation, the cartilage recovery of the SD rats in the PCL/PEG-Nar group was better than that in the PCL/PEG/Nar group and the PCL/PEG group. The results of H&E staining and safranin O/fast green staining indicated the smoothest and integrated cartilage structure and increased GAG content by PCL/PEG-Nar treatment. The superior therapeutic effect is attributed to the pH-responsive and sustained drug release without the need of repeated injection and systemic side effects. As a result, such biocompatible and biodegradable nanofibrous membranes may be loaded with drugs or therapeutic agents for synergistic therapy.

## Conclusion

In this study, we constructed a pH-sensitive PCL/PEG-Nar nanofiber membrane through the electrospinning method and demonstrated the therapeutic effect for OA treatment. PEG-Nar was successfully synthesized, and its chemical structure was verified by ^1^H NMR, FTIR, and ultraviolet absorption spectroscopy. The PCL/PEG-Nar nanofiber membrane had good mechanical and biological properties and realized controlled drug releasing under different pH conditions. *In vitro* and *in vivo* studies show that PCL/PEG-Nar nanofibers can inhibit the damage of inflammatory factors on chondrocytes, protect chondrocytes, and promote the regeneration of cartilage. The anti-inflammatory ability of the PCL/PEG-Nar nanofiber membrane is better than that of the PCL/PEG/Nar nanofiber membrane, which confirmed that Nar can be sustainably released in the weak acid environment of the osteoarthritis model. Despite having an excellent therapeutic effect, there are still questions, such as the interaction of scaffolds with biological systems, proper degradation rate, and long-term toxicity, which have to be further considered for the applications of the pH-sensitive electrospinning nanofiber membrane in clinic. In general, the PCL/PEG-Nar nanofiber membrane DDS provides a potential direction for the innovation of osteoarthritis treatment and provides a new theoretical basis for osteoarthritis treatment materials.

## Data Availability

The original contributions presented in the study are included in the article/Supplementary Material, further inquiries can be directed to the corresponding authors.
